# Anisole hydrodeoxygenation over Ni–Co bimetallic catalyst: a combination of experimental, kinetic and DFT study†

**DOI:** 10.1039/d2ra05136b

**Published:** 2022-10-26

**Authors:** Adarsh Kumar, Meenu Jindal, Shivam Rawat, Abhisek Sahoo, Rahul Verma, Devesh Chandra, Sagar Kumar, Bhaskar Thallada, Bin Yang

**Affiliations:** Bioproducts, Sciences, and Engineering Laboratory, Department of Biological Systems Engineering, Washington State University Richland WA 99354 USA bin.yang@wsu.edu; Academy of Scientific and Innovative Research, Kamla Nehru Nagar Ghaziabad 201002 India tbhaskar@iip.res.in; Material Resource Efficiency Division, CSIR-Indian Institute of Petroleum Dehradun 248005 India; Department of Chemical Engineering, Indian Institute of Technology-Delhi New Delhi 110016 India; Department of Chemistry, Indian Institute of Technology Kanpur Kanpur 20816 India; Chemical Technology Division, CSIR-Institute of Himalayan Bioresource Technology Palampur HP 176 061 India

## Abstract

Catalytic hydrodeoxygenation (HDO) of anisole was performed with a series of Ni and Co containing catalysts with different weight ratios on activated carbon (AC) for cyclohexanol production. The catalytic activities of various catalysts revealed that Ni_5_Co_5_-AC was the best catalytic system. Structural analysis obtained from XRD, TPR, XPS, and TEM evidently demonstrates that Ni_5_Co_5_-AC sample consists of a distorted metal alloy spinel structure and optimum particle size, enhancing its catalytic performance. Kinetics were investigated to identify cyclohexanol production rate, activation energy, and reaction pathway. Structural, experimental, kinetics and density functional simulations suggested that high amount of distorted metallic alloy in Ni_5_Co_5_-AC, presence of water, high adsorption efficiency of anisole, and low adsorption tendency of cyclohexanol on metallic alloy surface were the critical factors for HDO of anisole to cyclohexanol.

## Abstract 2

High reducible distorted bimetallic sites with medium size in Ni_5_Co_5_-AC promoted the production of cyclohexanol by hydrogenation of anisole and subsequent cleavage of C_6_H_11_O–CH_3_ bond.

## Graphical abstract

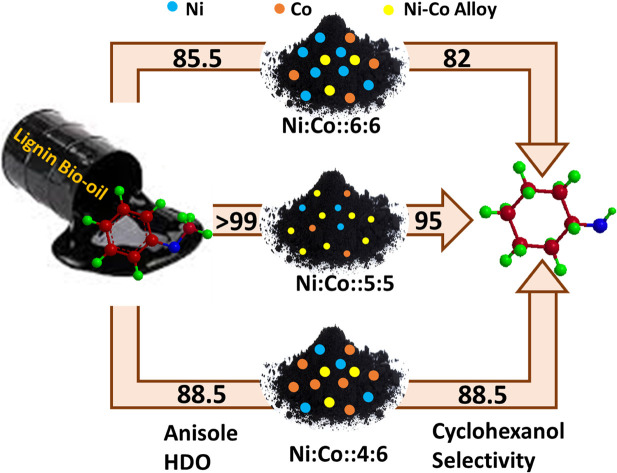

## 1. Introduction

Selective transformations of lignin derived compounds into aliphatic cyclic alcohols are attractive and fascinating for the future because these bio-based aliphatic cyclic alcohols are building blocks of amine derivatives, carboxylic acids, ketones, resins, polymers, paints, fine chemicals, and rubber.^1^ However, the selective hydrodeoxygenation process to produce cyclic alcohol is challenging. It includes the hydrogenation of unsaturated functional groups such as C=C and C=O and rupturing of O–C bond.^2^ Conventional hydrogen and liquid organic hydrogen carriers (LOHCs) can be used for HDO.^3^ However, noble metal catalysts, high temperature and uncontrolled HDO limit the use of LOHCs for low cost selective production of cyclic alcohol.^4–6^

Non-noble metal-based catalytic systems with high hydrogenation capability and controlled deoxygenation are critical for developing an efficient process.^7–10^ Literature reports suggest that the combination of Ni with transition metals such as Fe, Mo, Co, and Cu is more active and selective for HDO under ambient reaction conditions.^3,4^ Therefore, numerous bimetallic catalysts Ni–Co, Ni–Fe, Ni–Cu, and Ni–Mo have been used for HDO. It was found that Ni–Mo and Ni–Fe based catalysts are suitable for the production of aromatic compounds.^5,6^ Huynh *et al.*^11^ compared the activity of Ni–Co/ZSM-5 and Ni–Cu/ZSM-5 for phenol HDO and identified Cu incorporation to Ni deteriorated the catalytic performance owning to variable and large sized Cu species. At the same time, Co increased the activity along with selectivity. Co insertion promoted small and strongly stabilized active metal alloy sites with outstanding H_2_ activation and dissociation capability. Similarly, Liu and co-workers identified Co to be highly active for the selective cleavage of C_aryl_–OCH_3_ bond.^12,13^

Metal compositions significantly affect the textural property, reducibility, and acidity of the Ni–Co bimetallic^14^ catalysts. These changes are reflected in their catalytic performances. Ni–Co nanoparticle decorated porous Carbon/ZrO_2_ with different ratios of metals were used for phenol conversion.^15^ Ni_3_Co_1_@C/ZrO_2_ (15 wt%) showed the highest conversion (96%) and cyclohexanol yield (91%). The Ni–Co interaction and formation of bimetallic alloy were main causes for high activity. Zhou *et al.*^16^ investigated the hydrodeoxygenation of guaiacol over Ni–Co bimetallic systems with different ratios of Ni and Co. The equal metal loadings showed better activity than other compositions, taking temperature, hydrogenation and catalyst acidity into consideration. In another attempt, same research group investigated the reasons of high activity and reported that proper acidity, good metal dispersion and interaction between metal particles and support were responsible for it.^14^ Similarly, Li *et al.*^17^ investigated the impact of Co/Ni molar ratio (1 : 0 to 0 : 1) by changing incorporation amount of Ni and Co. Cyclohexanol amount in reaction system was increasing with Ni content, became maximum at 1Co–1Ni and then started decreasing. Authors claimed lowest alloy particle size to be a crucial factor for such high activity. Graphite supported bimetallic Ni–Co catalysts demonstrated improvement in reducibility, smaller particle size, and stability than monometallic catalyst.^18^ High Co containing catalyst led to higher selectivity towards phenol, while Ni_3_Co_1_ was the most active for cyclohexanol (≈65%) production with ≈80% guaiacol conversion. Very recently, Wang and co-workers^19^ compared the activity of Ni_*x*_Co_*y*_ bimetallic catalysts (Ni_*x*_ : Co_*y*_ = 1 : 0, 2 : 1, 1 : 1, 1 : 2, 1 : 3, 0 : 1) for cyclohexanol production. Results suggested that highest synergistic effect between Ni and Co, surface area and uniformly dispersed Ni–Co bimetallic sites in NiCo(1/2)@C-600 lead selectivity.

Although mentioned literature shows some promising signs for hydrodeoxygenation progress for cyclohexanol production, however, in-depth understanding of kinetic and mechanisms of the process and appropriate explanation of real time behaviour of Ni–Co bimetallic catalyst is still lacking. It is very crucial for a step forward to tune an efficient and engineered Ni–Co bimetallic catalytic systems with high hydrogenation capability and controlled deoxygenation activity for industry relevant cyclohexanols production. To contribute in this direction, herein, we developed Ni–Co nanoparticles impregnated activated carbon systems for selective HDO of anisole. The main objectives of this study are to understand effects of metal composition on active sites, and their role according to kinetic aspects with mechanistic pathways. The optimized catalyst showed unique behavior in both hydrogenation activity and deoxygenation selectivity. The kinetic and theoretical models were investigated to understand Ni–Co behaviour in real time reaction system to validate the mechanistic pathways for anisole hydrodeoxygenation.

## 2. Material synthesis and method

### 2.1. Materials

Activated carbon, ethyl acetate and other solvents were purchased from Fischer Scientific (≥99% purity). Anisole, and metal precursors, all are ≥98% purity, were purchased from Sigma-Aldrich and were used as received without any further treatment. The used double distilled water was synthesized in lab.

### 2.2. Catalyst synthesis

Activated carbon supported 10 wt% Ni, and Co catalysts were prepared by simultaneous co-impregnation method according to our previous study.^1^ The different loadings of Ni and Co were done by dissolving the calculated amount of Ni(NO_3_)_2_·6H_2_O and Co(NO_3_)_2_·6H_2_O in water (slightly in excess of the pore volume of the AC). The resultant slurry was stirred at room temperature with 200 rpm for 1 h then it was stirred at 80 °C for 2 h. The resultant catalysts were dried overnight at 105 °C, subsequently calcined at 325 °C in the presence of continuous nitrogen flow (100 mL min^−1^) with the 10 °C min^−1^ ramping rate for 2 h after reaching to the desired temperature. The calcined catalysts were reduced in continuous hydrogen flow (100 mL min^−1^) for 4 h at 600 °C. The reduced and powdered catalysts were used for characterization and catalytic activity.

### 2.3. Catalyst characterization

Prepared catalysts were characterized with various techniques such as powder X-ray diffraction (P-XRD), field emission scanning electron microscopy (FE-SEM) with energy-dispersive X-ray spectroscopy (EDS), transmission electron microscopy (TEM), N_2_-physisorption, H_2_-temperature programmed reduction (H_2_-TPR), NH_3_-temperature programmed desorption (NH_3_-TPD), Fourier-transform infrared spectroscopy (FT-IR), thermo-gravimeteric analysis (TGA) and X-ray photoelectron spectroscopy (XPS).

3Flex Physisorption, Micromeritics was used for N_2_-physisorption at liquid nitrogen temperature (−196 °C) to calculate the surface area, pore-volume, and pore size of the catalysts. Prior to the analysis, the surface of prepared catalysts was cleaned at 350 °C for 4 h under 1 × 10^−5^ torr vacuum. Brunauer–Emmett–Teller (BET) equation was used to determine the specific surface area (*S*_BET_) from the obtained adsorption data (*P*/*P*_0_ = 0.05–0.25).^20^ The average pore size was reported as a maxima of pore size distributions (PSDs) and these PSDs were evaluated from Barrett–Joyner–Halenda (BJH) algorithm using nitrogen adsorption branch data.^21^ The pore volumes of as prepared catalysts were measured as adsorbed volume of liquid nitrogen at *P*/*P*^0^ ≈ 1.

H_2_-TPR, chemisorption and NH_3_-TPD were employed on Micromeritics, Auto Chem II-HP 2920 in a U shaped quartz tube reactor equipped with a thermal conductivity detector (TCD). Prior to H_2_-TPR, chemisorption and NH_3_-TPD, the samples were heated with 10 °C min^−1^ in Ar and He, respectively to 300 °C for pre-treatment and temperature was maintained for 1 h. The pre-treated samples were cooled to 50 °C and analysis was done in the temperature range of 50–900 °C with a heating rate of 10 °C min^−1^. For NH_3_-TPD, the 50 mL min^−1^ flow of 10% NH_3_–He for 30 min was used for adsorption of NH_3_ on pretreated samples. The NH_3_ adsorbed samples were purged with pure He (20 mL min^−1^) for 30 min to remove the extra NH_3_–He. Subsequently, the temperature of prepared sample was raised to 900 °C and desorbed NH_3_ molecules were monitored on TCD. For H_2_-TPR, the analysis of pre-treated samples (non-reduced) was performed in 10% H_2_–Ar for the temperature range of 50–900 °C. IPA-liquid nitrogen slurry was used for condensation of released water vapors in the trap area, and quantity of adsorbed H_2_ was recorded in the form of TPR signals on TCD.

XRD patterns of powder samples were recorded in the range of 2*θ* = 2–80° on a Bruker D8 advance X-ray diffractometer fitted with a Lynx eye high-speed strip detector. The Cu Kα (*λ* = 1.5418 Å) used as a radiation source, while the scan rate was 0.02 step per sec for all samples. Shimadzu DTG-60 was used for TGA of catalysts. For TGA, the samples were heated in alumina cell with 10 °C min^−1^ under inert atmosphere (100 mL min^−1^ flow of N_2_) from room temperature to 900 °C. FT-IR of samples (prepared with KBr) were recorded in the range of 400–4000 cm^−1^ using FTIR-Perkin Elmer-Spectrum II instrument.

TEM/HRTEM was performed on JEOL JEM 2100 microscope. The images were taken by mounting an ethanol-dispersed sample on a lacey carbon formvar Cu grid at an operating voltage of 200 kV. Elemental mapping was also recorded using same spectrophotometer and EDS was used for the elemental composition of samples. SEM images were taken with FEI Quanta 200 F, having tungsten filament as an X-ray source doped in lanthanum hexaboride (LaB_6_), fitted with an ET (Everhart–Thornley) detector, using secondary electrons and an acceleration tension of 10 or 30 kV in high vacuum.

XPS for the surface study of Ni–Co catalyst was performed, using ESCA+, (omicron nanotechnology, Oxford Instrument Germany) equipped with monochromator aluminum source (Al ka radiation *hv* = 1486.7 eV). The instrument was operated at 15 kV and 20 mA. To overcome the charging problem, a charge neutralizer of 2 keV was applied, and binding energy of C 1s core (284.6 eV) was taken as reference. High-resolution XPS (HR-XPS) was collected by passing energy of 69.0 eV with a step size of 0.125 eV. The HR-XPS profiles of Ni and Co were fitted using a Gaussian function, and peak positions were normalized corresponding to the C 1s (284.6 eV).

### 2.4. Catalytic activity

The catalytic activity of various catalysts was performed in a 30 mL customized stainless steel tubular reactor equipped with a pressure gauge. The 100 mg reduced catalyst, 0.5 mmol anisole and 5 mL water were loaded in the reactor. The reactor was purged thrice with 1 MPa hydrogen for generating hydrogen environment and then pressurized to desired hydrogen pressure. The pressurized reactor was fitted to the pre-heated silica oil bath with magnetic stirring at 1000 rpm for the reaction time. The reactor was depressurized after cooling in water bath and organic fraction was collected through liquid–liquid separation. Ethyl acetate (15 mL) was used for extraction of organic phase and ethyl acetate was evaporated from organic mixture in rotatory evaporator. The obtained organic fraction was diluted with 3 mL ethyl acetate and collected in 5 mL vial.

The qualitative analysis of the organic fraction was performed in Agilent GC (7890B) equipped with a mid polar capillary column (DB-35MS, 35% phenyl/65% dimethylpolysiloxane, 30 m × 0.32 mm × 0.25 μm) connected to a mass spectrometer (5977A MSD). The quantitative analysis of the product was performed on Shimadzu GC-FID (GC-2014) equipped with RXi-MS (100% dimethylpolysiloxane, 30 m × 0.25 mm × 0.25 μm) capillary column. *n*-Dodecane was used as internal standard for quantification. Conversion and selectivity is reported as the average of two reactions, performed at the same experimental conditions to minimize experimental uncertainty. The conversion and selectivity are reported with ±2% standard deviation and calculated as follows1
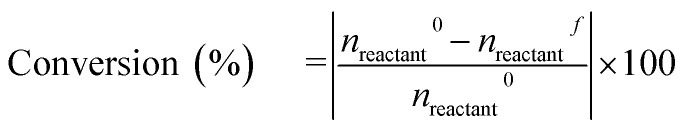
2
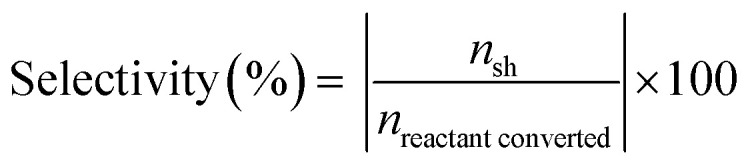
where, *n*_reactant_^0^ is the initial concentration of reactant and *n*_reactant_^*f*^ is the concentration of reactant after reaction in mole per litre. The *n*_sh_ denotes the mol per litre concentration of selective hydrodeoxygenated product.

### 2.5. Computational modelling

To model the catalytic reactions, spin-polarized periodic density functional theory (DFT) calculations were carried out using Quantum Espresso (QE) package.^22,23^ The PBE (Perdew–Burke–Ernzerhof) exchange–correlation density function^24^ was used in the calculations, while the core electrons were represented by the Vanderbilt ultrasoft pseudo potential.^25^ The Kohn–Sham orbitals were expanded with plane-wave basis sets with a kinetic energy cutoff of 300 eV, and the *k*-point sampling used for the integration in reciprocal space was limited to Gamma-point only. The experimental characterization revealed that the catalyst has a chemical composition of Ni_5_Co_5_-AC and the formation of (111) surface. Thus, to account for the metal composition effects on the reaction path, a 4-layered 3 × 3, Ni–Co (111) bimetallic surface (Ni_72_Co_72_) was created as shown in Fig. 5. In our model, the activated carbon support was not considered since it does not take part in the catalytic reactions. A vacuum of 15 Å was added along the *Z*-axis to minimize the interaction of the surface with its own replica. The structures were optimized using the BFGS algorithm^26^ as implemented in the QE package, where the top two out of four atomic layers of the 3 × 3 Ni–Co (111) surface were relaxed, whereas the atoms in the lower two layers were kept frozen to the bulk lattice position.

The adsorption energies (Δ*E*) of all the species, which could form during the HDO process, were calculated according to the following equation,

3 Δ*E* = *E*_system_ − (*E*_adsorbent_ + *E*_adsorbate_)

where, *E*_system_ is the total energy of adsorbed species on the Ni–Co surface, while *E*_adsorbent_ is the energy of adsorbent molecules (*e.g.* anisole, cyclohexanol, and methoxycyclohexanol (MCH)), and *E*_adsorbate_ is the energy of bare NiCo surface.

## 3. Results and discussion

### 3.1. Catalyst characterization

**Fig. 1:**
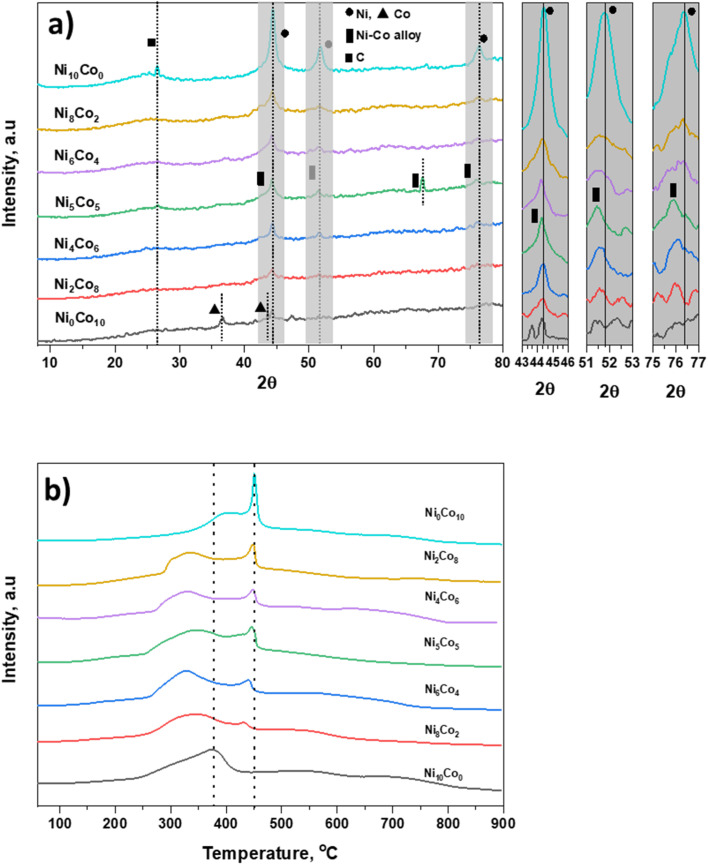
(a) P-XRD pattern; (b) H_2_-TPR profile of activated carbon supported NiCo-AC catalysts.

Physical characteristics of catalysts are given in Table S1.† Table states that surface area and pore volume of AC are 938 m^2^ g^−1^ and 0.67 cm^3^ g^−1^, respectively. In all prepared catalysts, there is decrease in surface area and pore volume due to deposition of metal particles.^27^ There is no drastic difference in surface areas of all prepared catalysts. XRD patterns of activated carbon supported Ni and Co catalysts are shown in Fig. 1a. A broad peak in the region 21–29° centred around at 26° is attributed to carbon. The Ni_10_Co_0_-AC and Ni_0_Co_10_-AC show the typical diffraction patterns corresponding to Ni and Co metal.^28,29^ The most intense diffraction peaks of Ni and Co are located at 2*θ* value of 44.4° (111), and 44.2° (111), respectively.^14^ These characteristic peaks of Ni and Co do not precisely match with the Ni–Co bimetallic catalysts. The peak of Ni–Co bimetallic catalyst shifted corresponding to Ni–Co alloy (111), can be seen in inset figure between 43 and 46°.^30,31^ The other signature peaks of nickel phase (51.8°, and 76.3°) also shift towards lower 2*θ* values (51.5° and 75.8°) with reduced intensities associated with the Ni–Co alloyed phase (200) and (220),^30,32^ respectively. All these peaks reposition from their original positions, suggesting the formation of Ni–Co alloy due to the metal–metal interaction. Besides this, an additional peak can be seen at 67.5° corresponding to Ni–Co alloy phase in Ni_5_Co_5_-AC.^30^ Therefore, Ni–Co metal alloy formation in the Ni–Co bimetallic catalyst depends on the Ni/Co ratio. These results are well in accordance with the previous literature reports, which suggested the existence of the characteristic diffraction peak in the region (43–47°) and showed the peak shift in between 51–53° and 75–77° for the supported Ni–Co bimetallic catalysts due to the formation of Ni–Co alloy phase.^30,33–35^

H_2_-TPR of all catalysts was employed for the identification of the reducibility of catalyst and the specific interaction between metal species and support (Fig. 1b). TPR spectra of Ni_10_Co_0_-AC consists of one broad reduction peak in the range of 250 °C to 425 °C, centred at 375 °C. TPR spectra of Ni_0_Co_10_-AC consists one large peak at 450 °C with a shoulder peak at 296 °C, it reflects two-step reduction of Co_3_O_4_ to CoO and subsequent CoO reduction to Co.^36^ High reduction temperature of Ni_0_Co_10_-AC is suggesting a strong interaction between Co species and carbon support. There are two peaks in the mixed oxides. In mixed oxides, a shift in parent peaks to lower temperatures in comparison of Ni_10_Co_0_-AC and Ni_0_Co_10_-AC indicates that the interaction of Ni and Co atoms in bimetallic catalysts would contribute to the improvement of reducibility of catalysts.^37,38^ This type of interaction has been well reported in literature for Ni/Co catalysts which lead alloy formation on the surface of the support.^34,39^ This peak is at lowest temperature in Ni_5_Co_5_-AC and suggests that this particular sample is highly reducible due to high interaction between Ni and Co. With this point and according to XRD, and TEM results the formation of mixed spinal phase and high dispersion of metals in Ni_5_Co_5_-AC are confirmed.

**Fig. 2:**
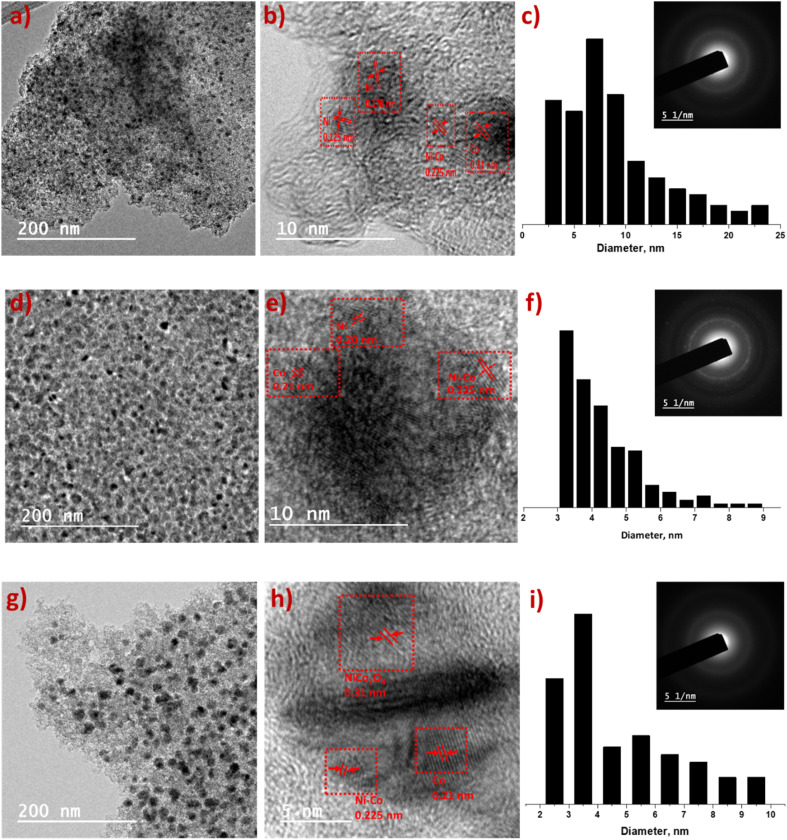
HR-TEM images of Ni_6_Co_4_-AC (a–c), Ni_5_Co_5_-AC (d–f) and Ni_4_Co_6_-AC (g–i). (b, e and h) interplanar *d*-spacing in high magnified TEM; (c, f and i) particle size range of metals and SAED pattern.

Surface texture and microstructure of the synthesized materials were checked using HR-TEM. High magnified TEM images (Fig. 2b, e and h) indicate Ni, Co and mixed metal nanoparticles are embedded on AC surface. The dark spots in TEM images (Fig. 2a, d and g) of catalysts and TEM-EDS mapping (Fig. S1†) show homogeneous distribution of metal nanoparticles. Ni_6_Co_4_-AC, Ni_5_Co_5_-AC and Ni_4_Co_6_-AC demonstrate common lattice distances 0.225 nm and 0.21 nm corresponding to (111) plane of Ni–Co alloy and cobalt metal.^40,41^ Ni_6_Co_4_-AC shows two types of inter planar distances for (111) and (220) planes of Ni validating high Ni amount.^42^ Similarly, Ni_4_Co_6_-AC surface texture (Fig. 2e) shows cobalt, alloy and mixed oxides (220) phases.^43^ SAED pattern of Ni_5_Co_5_-AC represents somehow crystallinity possibly due to high content of Ni–Co alloy. Measured metal particles range and average size (Fig. 2c, f and i) of the Ni_6_Co_4_-AC, Ni_5_Co_5_-AC and Ni_4_Co_6_-AC catalysts suggest that cobalt promotes the size reduction and dispersion.^44^ Average size was minimum (3.2 ± 0.2 nm) for Ni_5_Co_5_-AC and is in consistent with literature.^17,44,45^

**Fig. 3:**
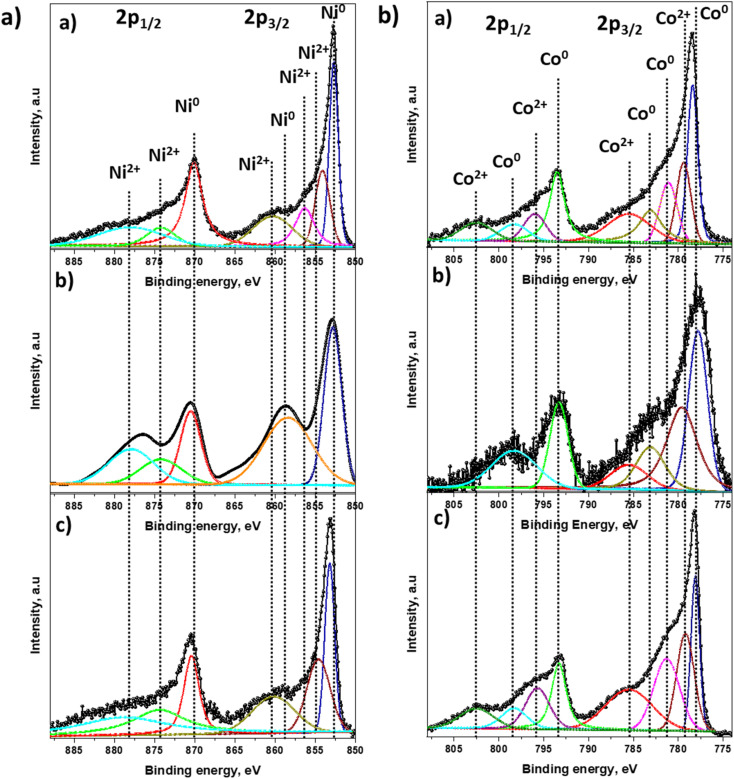
HR-XPS spectra of (a) Ni 2p; (b) Co 2p. Insight (a)–(c) represents Ni_6_Co_4_-AC, Ni_5_Co_5_-AC and Ni_4_Co_6_-AC.

Chemical state and surface element composition of Ni_6_Co_4_-AC, Ni_5_Co_5_-AC and Ni_4_Co_6_-AC catalysts were analyzed by XPS to further identify the interaction between Ni–Co. The peak intensity of Ni and Co in survey scan indicates Ni and Co amounts in catalysts (Fig. S2†). In HR-XPS graphs of Ni 2p, the binding energies of Ni 2p_3/2_ and Ni 2p_1/2_ are positively shifted (Fig. 3a) from original peaks (852.4 and 870.1 eV).^14^ Likewise in the Co 2p spectra (Fig. 3b), the peak at 777.8 (Co 2p_3/2_) is negatively shifted by 0.4 eV of Co (778.1 eV), however it arises at its original position in Ni_4_Co_6_-AC that might be due to the high amount of Co.^14,46^ These peak shifts reveal the electronic interactions between Ni and Co and formation of Ni–Co alloy.^46^ Ni 2p_3/2_ and Co 2p_1/2_ of Ni_5_Co_5_-AC are well-fitted with two doublets of Ni^0^ and Co^0^ suggesting that easily reducible nature of Ni_5_Co_5_-AC and presence of high amount of Ni–Co alloy than Ni_6_Co_4_-AC and Ni_4_Co_6_-AC. These results are well aligned with XRD, TPR and TEM results.

### 3.2. Prodigious effects of reaction parameters

**Fig. 4:**
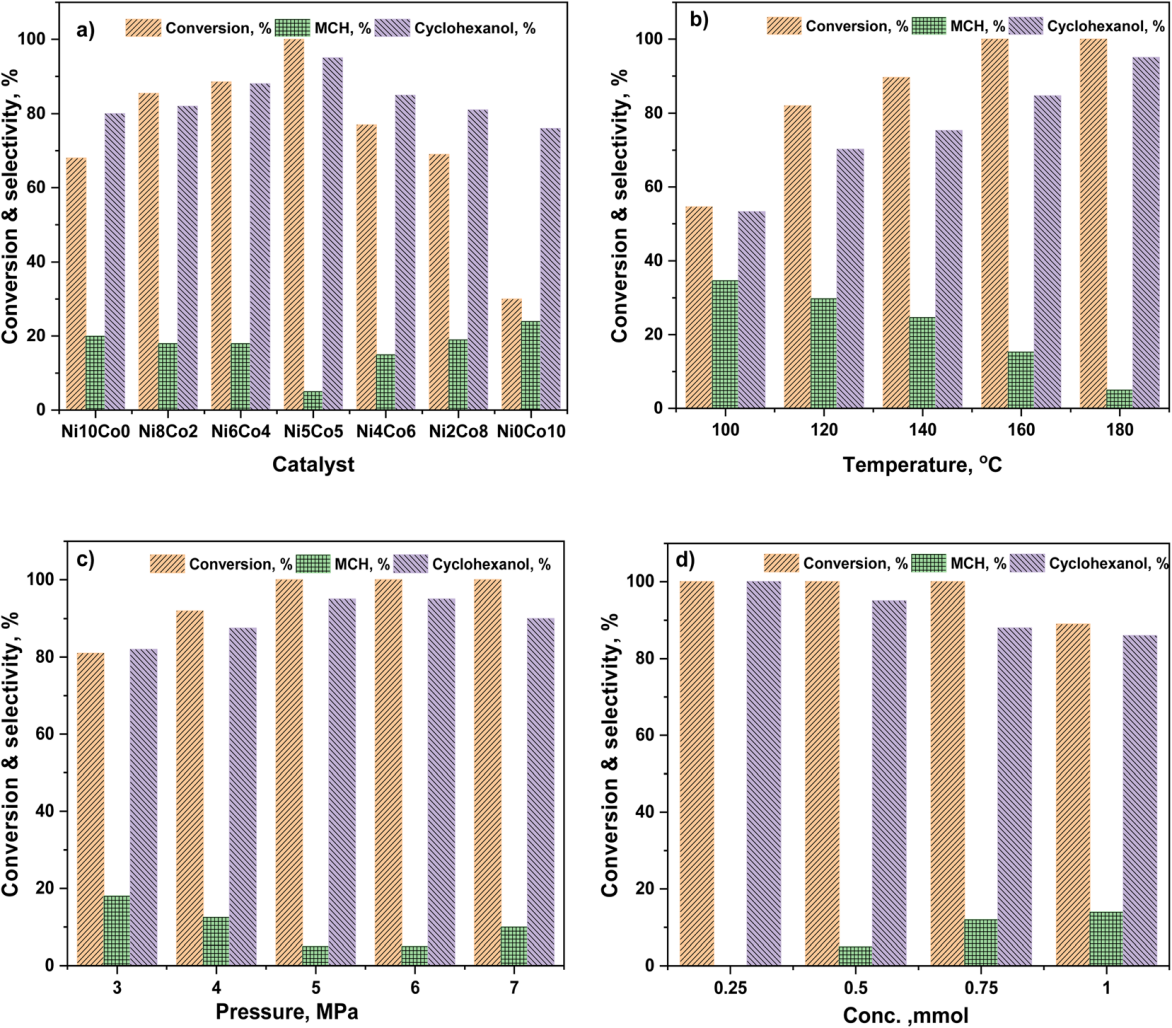
Anisole hydrodeoxygenation; (a) effect of catalyst composition, (b) effect of temperature, (c) effect of pressure, (d) effect of feed concentration. Reaction conditions: anisole – 0.5 mmol, catalyst – Ni_5_Co_5_-AC (100 mg), *T* – 180 °C, *P* – 5 MPa, water – 5 mL and time – 4 h.

Anisole conversion of 30% with MCH (76%) and cyclohexanol (24%) as the primary products was observed over Ni_0_Co_10_-AC catalyst. In contrast, the anisole conversion was 68% and selectivity of cyclohexanol and MCH was 80% and 20%, respectively, over Ni_10_Co_0_-AC. The high conversion of anisole over Ni_10_Co_0_-AC demonstrated the high hydrogenation ability of metallic Ni than Co. Anisole conversion and product distribution over monometallic catalysts (Ni_10_Co_0_-AC and Ni_0_Co_10_-AC), and bimetallic catalysts (NiCo-AC) with various Ni/Co ratios are compared to reveal the bimetallic effect in Ni–Co catalyst (Fig. 4a). As shown in Fig. 4a, the presence of Ni with Co in a single catalyst remarkably affects both the activity and selectivity of the catalyst. With the introduction of nickel content, the NiCo_2_O_4_ phase is formed due to insertion of Ni^2+^ ions into octahedral sites of Co_3_O_4_ having spinel structure, and NiCo_2_O_4_ changed into Ni–Co alloy during reduction. Ni/Co ratio strongly influences the formation of these active surface species and their structure as well as the activity of Ni_*x*_Co_*y*_-AC catalysts. Structural distortion in Ni–Co alloy increases with increase in nickel content due to maximum insertion of Ni into octahedral sites.^47–49^ Ni–Co bimetallic sites in Ni_*x*_Co_y_-AC works synergistically with the monometallic active sites, and increase the hydrogenation ability.^47^ The high selectivity towards cyclohexanol highlights the critical role of neighbouring Ni–Co bimetallic active sites for the adsorption of anisole and subsequent cleavage of C_6_H_11_O–CH_3_ bond. Anisole conversion increased to 69% over Ni_2_Co_8_-AC with 81% cyclohexanol selectivity. High amount of Ni–Co alloy with the highest distortion and small size led complete conversion of anisole over the Ni_5_Co_5_-AC catalyst, and selectivity towards cyclohexanol (95%) (Fig. 4a). XRD, TEM, H_2_-TPR, and XPS results attest to this fact. A significant drop in anisole conversion (11.5%) and cyclohexanol selectivity (7%) was observed over Ni_6_Co_4_-AC in comparison to Ni_5_Co_5_-AC might be owing to low amount of bimetallic sites and large size of metals in Ni_6_Co_4_-AC.

Solvent has a significant effect on anisole conversion and product distribution as observed in various studies.^50–52^ The activity order of Ni_5_Co_5_-AC for anisole conversion in various solvents is water > isopropanol > isobutanol > ethanol > methanol (Table S2†). The maximum conversion was 13%, and the major product was MCH (72%) when isopropanol was used as a solvent (entry 3, Table S2†). Results suggest that HDO was a slow reaction in alcohol, and a long reaction time is required for complete conversion (entry 4, Table S2†). However, a drastic change in conversion and product distribution was observed in water where conversion reached to >99%, and cyclohexanol was obtained as a primary product (95%). Low solubility of anisole in water promotes the ability of the reagent to adsorb on active catalyst sites due to less interaction between anisole and water, additionally, high polarity of water causes the hydrogenolysis of ArO–CH_3_ bond.^53,54^ This explanation is well confirmed by Yang and co-workers,^62^ who state that hydrogenation of the anisole is expected to take place through the π-complex formation, following to adsorption of anisole on active metal surfaces.

The reaction temperature played a significant role in the HDO of anisole.^55^ The effect of temperature (100–180 °C) for anisole HDO with product distribution over Ni_5_Co_5_-AC is shown in Fig. 4b. Anisole conversion is only 31%, however, cyclohexanol selectivity was 70.5% at 80 °C. The increase in temperature gradually increased anisole conversion and cyclohexanol selectivity. Conversion reached ≥99.9% at 180 °C, and selectivity for cyclohexanol was maximum (95%). On the basis of results, it can be assumed that at high temperature, catalyst exhibited more demethylation behavior due to scission of C_6_H_11_O–CH_3_ bond and cyclohexanol appeared as major product. It might be attributed to the lower bond dissociation energy of HO–CH_3_ (377 kJ mol^−1^ at STP) than H–OCH_3_ (436.8 kJ mol^−1^ at STP) and H–OH (498.7 kJ mol^−1^ at STP).^56,57^ Hydrogen pressure had an enormous effect on both anisole conversion and product distribution (Fig. 4c). Anisole conversion was 81% with 82% cyclohexanol selectivity at 3 MPa. Anisole conversion and cyclohexanol gradually increased with pressure and reached maximum (>99.9% and 95%) at 5 MPa and no significant change in cyclohexanol selectivity was observed above this pressure. The enhancement in anisole conversion and cyclohexanol selectivity at hydrogen pressure (≤5 MPa) can be clarified from two view points. First, hydrogen was a reactant in the experiment and increased pressure favours the hydrogenation reaction and scission of O–CH_3_ bond. Second, the solubility of hydrogen in water was high at high pressure, which would require improvement of hydrogen accessed on the catalyst active sites during the hydrogenation reaction.^58,59^ The impact of feed conc. can be seen in Fig. 4d. At the lower conc. (0.25 mmol), there was selective conversion towards cyclohexanol. As the conc. of feed was increasing, the selectivity of cyclohexanol was decreasing. It was due to that the reacting molecules were increasing while the active sites were constant. Reusability of catalyst is an important feature of heterogeneous catalysis for economic chemical transformation. In reusability, catalyst was separated by centrifugation after each catalytic run, washed with isopropanol to remove organic adsorbent from the catalyst surface and dried at 105 °C. Table S3† shows catalyst reusability results. The catalyst was losing activity in consecutive runs, it was owing to loss and agglomerations of active particles and metal oxide formation^60,61^ (Fig. S3†).

### 3.4. Mechanistic and kinetic study over Ni_5_Co_5_-AC

**Fig. 5:**
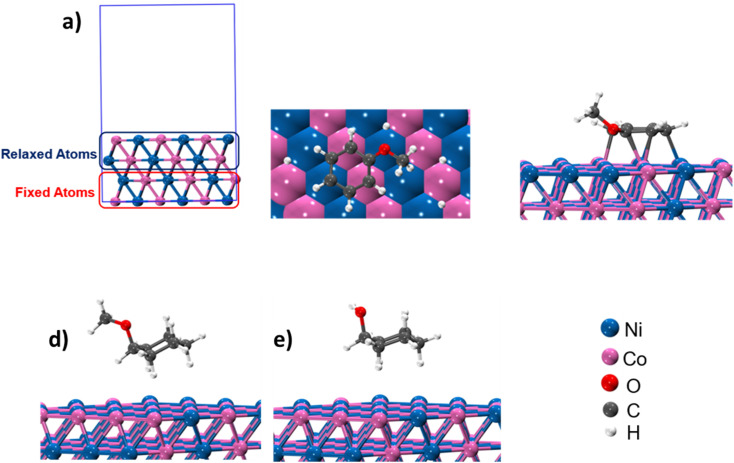
(a) and (b) Structure and different orientation of Ni_5_–Co_5_; (c)–(e) binding configuration of anisole, MCH and cyclohexanol.

Product distribution demonstrates only two products. MCH resulted from the aromatic ring hydrogenation of anisole, which might take place following the π-complex adsorption of the anisole on active sites of the catalyst.^62^ The possible reason for the appearance of cyclohexanol was weak HO–CH_3_ linkage.^56,57^ However, identification of dominant pathway of anisole transformation under hydrotreating conditions is complex.^56,63^ To understand this, time on stream data was recorded (Table S4†). Up to the 10 min reaction time, it was very hard to identify the dominant reaction path. However, MCH was the only product at the very initial stage (5 min). As the reaction time increased, cyclohexanol started appearing due to the demethylation of MCH. It suggests that anisole hydrodeoxygenation follows hydrogenation and subsequent demethylation pathway. DFT calculations were carried out to further validate the reaction mechanism and high selectivity of cyclohexanol. The experimental characterization revealed that the catalyst has a chemical composition of Ni_5_Co_5_-AC and the formation of (111) surface. Thus, to account for the metal composition effects on the reaction paths, a 4-layered 3 × 3, Ni–Co (111) bimetallic surface (Ni_72_Co_72_) was created (Fig. 5), considering catalyst support (activated carbon) as neutral and is not taking part in the HDO of anisole. Similar Ni–Co clusters have been used to study Congo red adsorption.^64^ Heterogenous catalysis follows catch release mechanism. Thus, the activity of Ni–Co cluster and product distribution depend on the strength of the adsorption of reacting and product molecules. The binding energies are listed in Table 1 for different reaction products including reactant and their binding configurations are shown in Fig. 5. The strong binding energy of anisole (−1.41 eV) is mainly due to the interaction of surface metal atom with the π cloud of the aromatic benzene ring. Since there are no π electrons in the cyclohexane ring, the interaction of the cyclohexanol and MCH molecules to the surface is weak. The binding energy calculations suggested that direct anisole hydrogenation to MCH is kinetically and thermodynamically favourable. It should be noted that MCH can either desorb or undergo further hydrogenolysis to cyclohexanol. MCH appearance in the product profile at very initial stage confirmed its spontaneous desorption on the Ni–Co (111) surface. In contrast, relative low adsorption energy of cyclohexanol assisted the fact of cyclohexanol as the primary product under optimum reaction conditions. The isotope labelling experiment results also suggest the same product distribution although there was deuterated cyclohexanol and MCH (Fig. S4†).

**Table 1:** Binding energy of reactant and product molecules in reaction on the 3 × 3 surface of Ni–Co (111)

Molecule	Adsorption energy, eV per molecule
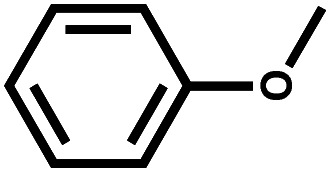	−1.14
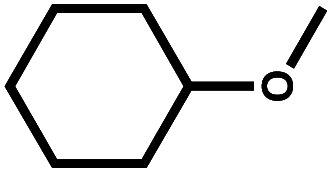	−0.43
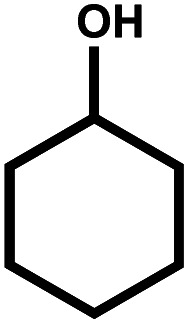	−0.41

**Fig. 6:**
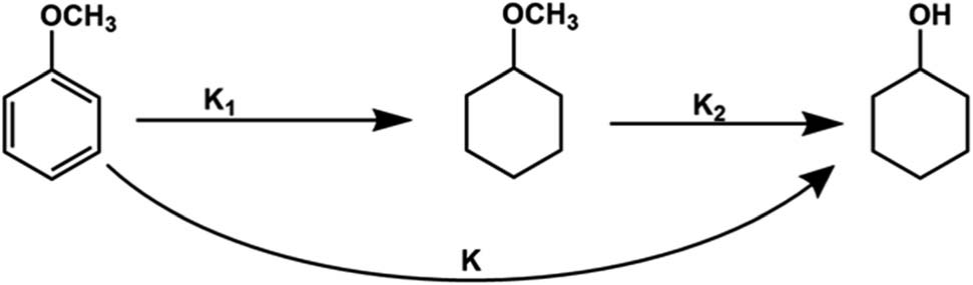
Reaction mechanism and pathways of anisole hydrogenation over Ni_5_Co_5_-AC.

For kinetics, reaction route can be simplified (Fig. 6) as an efficient direct reaction from anisole to cyclohexanol due to low selectivity of MCH than cyclohexanol (*i.e.*, *K*_1_ ≪ *K*_2_ = *K*). Moreover, there were approx 134 molecules of H_2_ for one molecule of anisole and assumed hydrogen in excess and was not considered during calculations.

According to the simplified route, the rate of anisole decomposition can be given as below:4
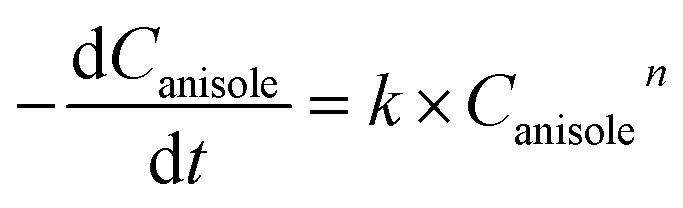
where, *k* and *n* represent the reaction rate constant and reaction order (*n* ≠ 1), respectively.

Integrate the eqn (4) to have5
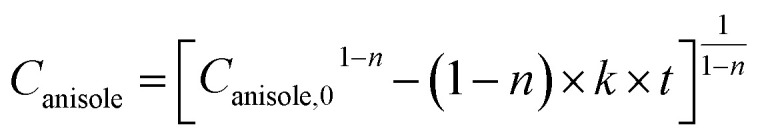
where, *C*_anisole,0_ represents the original concentration of anisole.

Thus, the real-time concentration of produced cyclohexanol, *C*_cyclohexanol_ can be described as follows, *i.e.*,6
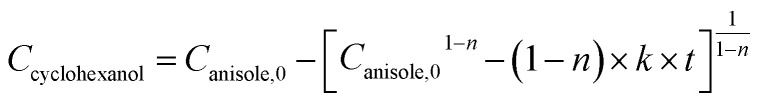
where, *C*_cyclohexanol_ represent the real-time concentration of produced cyclohexanol.

According to the Arrhenius equation, the reaction rate constant can be described as:7
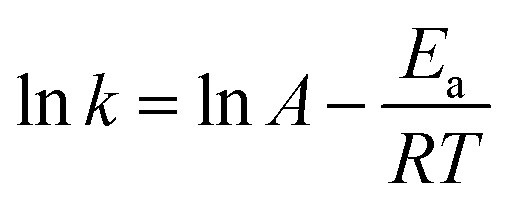
where, *E*_a_ and *A* represents the activation energy and frequency factor, respectively.

According to eqn (6) and (7), the real-time concentration of produced cyclohexanol can be written as8
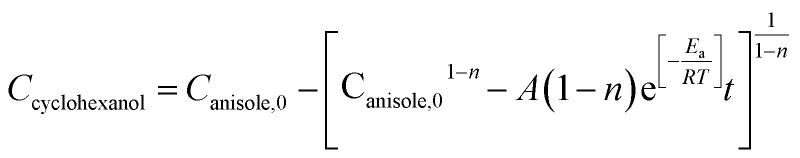


**Fig. 7:**
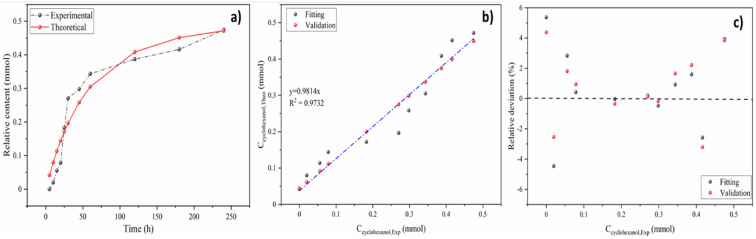
The correlation (a), validation (b), and relative deviation (c) between the predicted and experimental data.

The real-time concentration measurements of anisole at various temperatures and times were used to compute the parameters of reaction kinetics, *i.e. E*_a_, *A* and *n* shown in eqn (8). Calculated activation energy and frequency factor through established model were 33.49 kJ mol^−1^ and 154.38 min^−1^, respectively. The value of activation energy of cyclohexanol production for Ni_5_Co_5_-AC is much lower than monometallic Ni-based catalysts (48 and 56 kJ mol^−1^) and Ni_15_Co_5_@C/ZrO_2_ (44.31 kJ mol^−1^).^15,65^ The reaction order (1.26) is nearly 1, indicating anisole hydrodeoxygenation is a pseudo first-order process in a subcritical water medium. It is well aligned with reaction order (1.125) calculated by the initial rate method (ESI†). Fig. 7a illustrates the plot between experimental and theoretical values with respect to time (h). The theoretical and experimental values of *C*_cyclohe*x*anol_ have a strong connection (*R*^2^ = 0.97), as can be shown in Fig. 7b. Further examining the robustness of the created model required calculating the relative deviations (RD, %) between the fitting and validation values as shown in Fig. 7c. As can be observed, most RD data for predicted values are within 7%, suggesting that the current model can adequately describe the HDO of anisole.

## Conclusions

Results from NiCo-AC catalyst series with various Ni/Co ratios suggest that catalyst composition is a key factor in controlling selective anisole HDO to cyclohexanol. The insertion of Ni^2+^ ions into octahedral sites of Co_3_O_4_ spinel structure formed NiCo_2_O_4_, and the formed phase changed into Ni–Co alloy during the reduction. High reducible nature and medium particle size range (3–5 nm) of Ni_5_Co_5_-AC promoted the production of cyclohexanol. It highlights the critical role of neighbouring Ni–Co bimetallic active sites for the adsorption and hydrogenation of anisole and subsequent cleavage of C_6_H_11_O–CH_3_ bond. The hydrogenation pressure and reaction temperature affect both the anisole conversion and product distribution. The apparent hydrogen pressure and reaction temperature are 5 MPa and 180 °C for the Ni_5_Co_5_-AC catalyst. The mechanistic study revealed the reaction path and MCH appearance in the product profile at the very initial stage confirmed its spontaneous desorption from the Ni–Co (111) surface. However, low activation energy for cyclohexanol production and comparatively low binding energy of cyclohexanol assisted anisole HDO is kinetically and thermodynamically favourable. Based on the initial rate method and the established kinetics model, results indicate anisole hydrodeoxygenation is a pseudo-first-order process in a subcritical water medium.

## Conflicts of interest

There are no conflicts of interest.

## Supplementary Material

RA-012-D2RA05136B-s001
