# Supplementary material for: Anisole hydrodeoxygenation over Ni–Co bimetallic catalyst: a combination of experimental, kinetic and DFT study
Source: RSC Adv. 2022 Oct 26;12(47):30236–47. doi: 10.1039/d2ra05136b (PMC9597293; doi:10.1039/d2ra05136b)
Supplement: RA-012-D2RA05136B-s001 [file RA-012-D2RA05136B-s001.pdf]

Electronic Supplementary Material (ESI) for RSC Advances.
This journal is © The Royal Society of Chemistry 2022

Anisole hydrodeoxygenation over Ni-Co bimetallic catalyst: A combination of experimental, kinetic and DFT study

Adarsh Kumar [a], Meenu Jindal [b,c], Shivam Rawat [b,c], Abhisek Sahoo [d], Rahul Verma [e], Devesh Chandra [b,f], Sagar Kumar [c], Bhaskar Thallada [b,c*], Bin Yang [a*]

[a] Bioproducts, Sciences, and Engineering Laboratory, Department of Biological Systems Engineering, Washington State University, Richland, WA 99354, USA

[b] Academy of Scientific and Innovative Research, Kamla Nehru Nagar, Ghaziabad 201002, India

[c] Material Resource Efficiency Division, CSIR-Indian Institute of Petroleum, Dehradun 248005, India

[d] Department of Chemical Engineering, Indian Institute of Technology - Delhi, New Delhi, 110016, India

[e] Department of Chemistry, Indian Institute of Technology Kanpur, Kanpur 20816, India

[f] Chemical Technology Division, CSIR- Institute of Himalayan Bioresource Technology, Palampur, HP 176 061, India

Email: bin.yang@wsu.edu, tbhaskar@iip.res.in

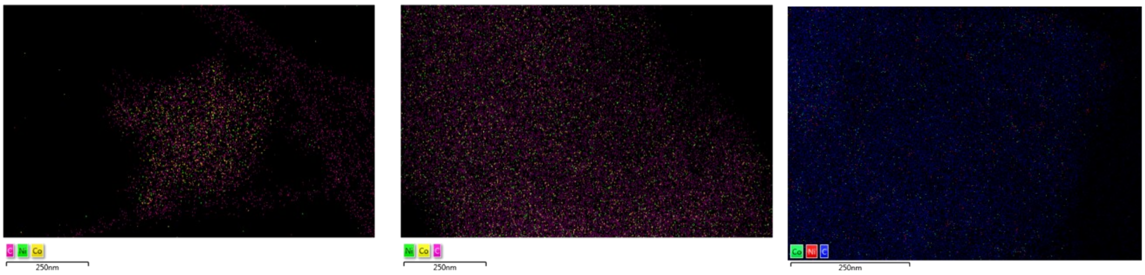

Figure S1: EDX mapping of Ni_6Co_4-AC, Ni_5Co_5-AC, and Ni_4Co_6-AC

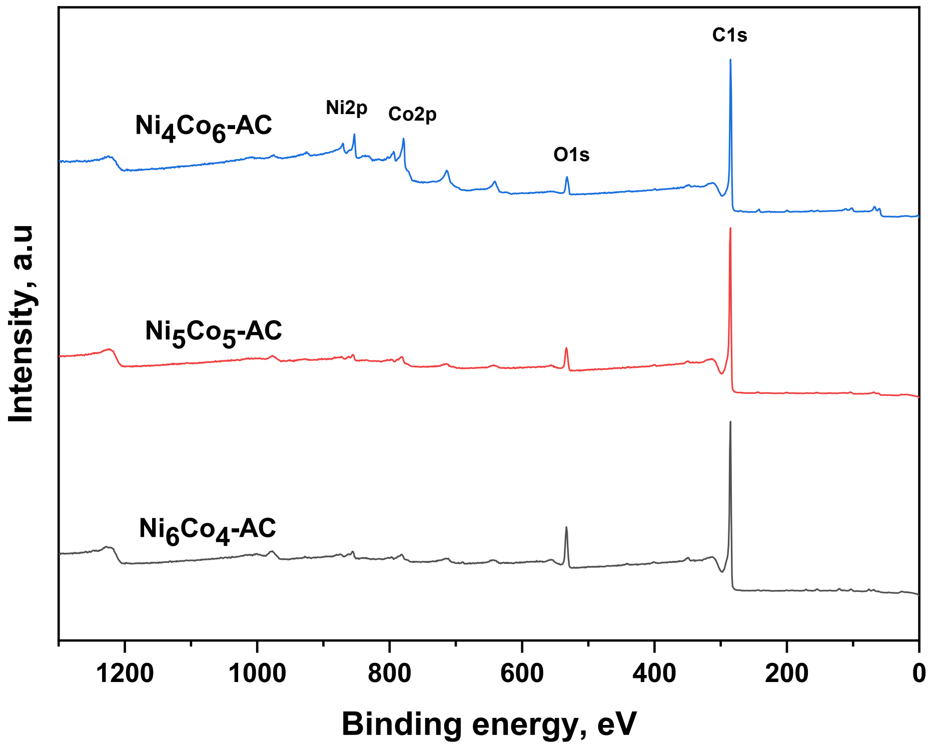

Figure S2: Survey scan of Ni_6Co_4-AC, Ni_5Co_5-AC, and Ni_4Co_6-AC

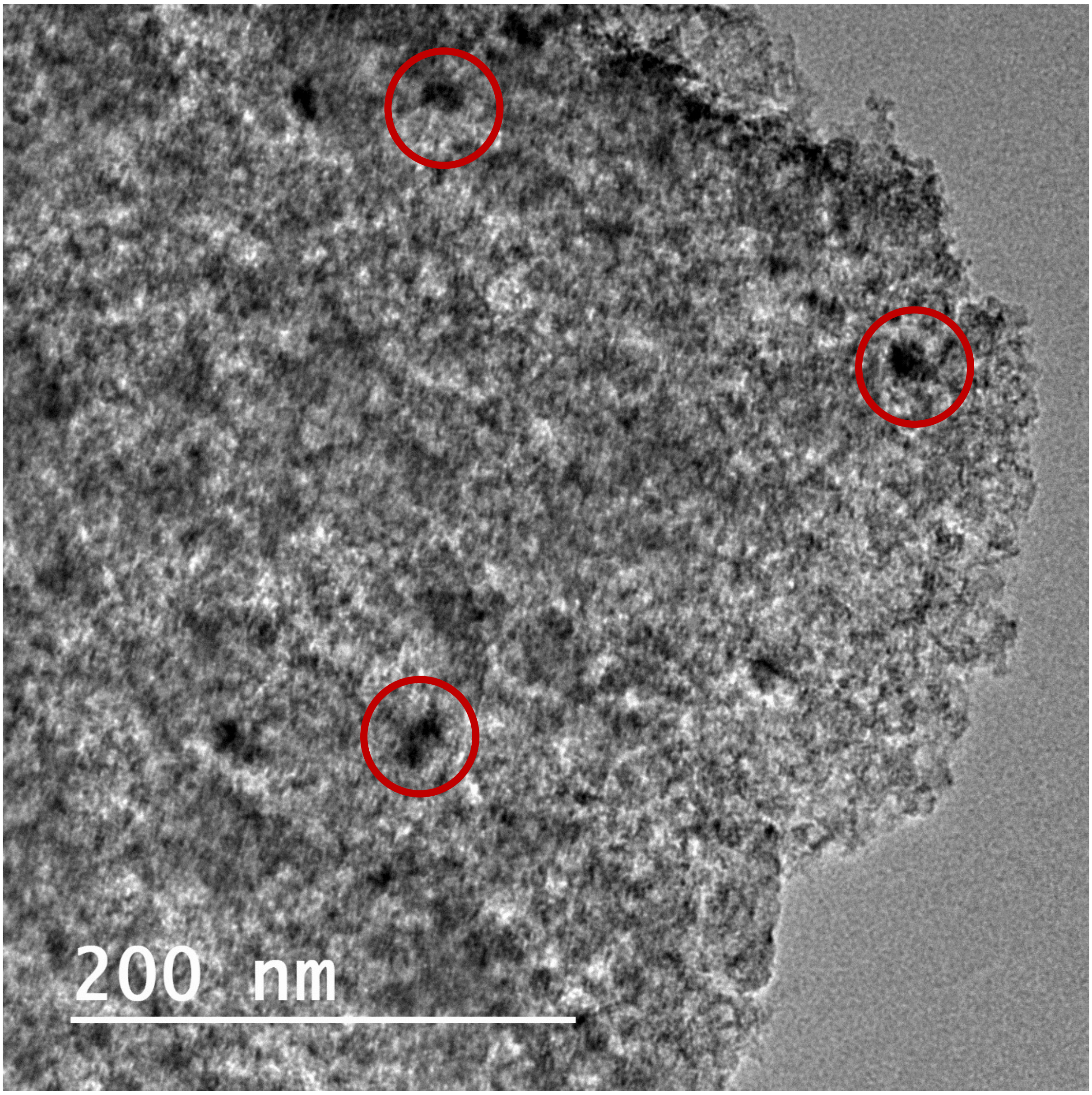

Figure S3: Agglorimation of Ni-Co in spent catalyst.

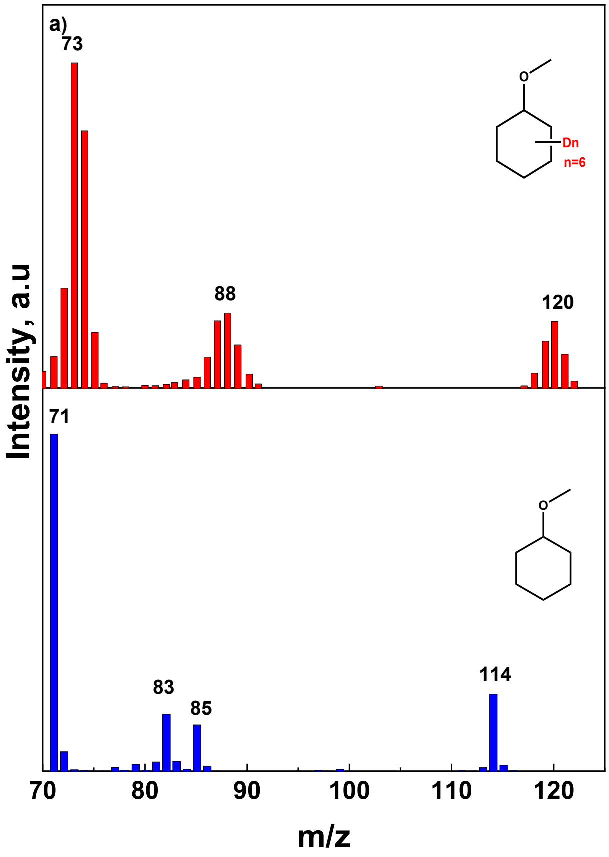

a)
73
88
120
Dn
n=6
o
71
83
85
114
o
Intensity, a.u
70
80
90
100
110
120
m/z

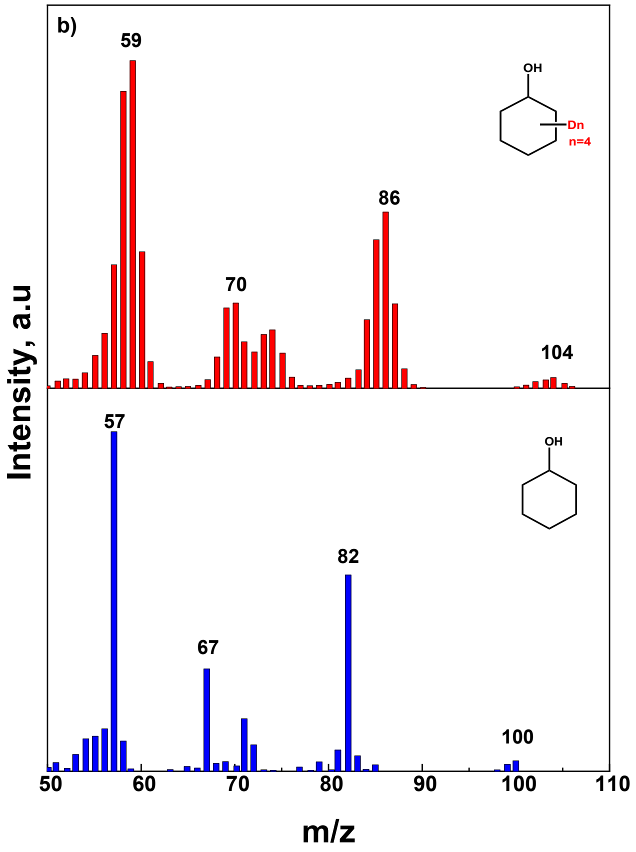

Figure S4: MS ion chromatogram of products of anisole HDO over Ni_5Co_5-AC in D_20; Reaction condition - Catalyst- 100mg, T-180°C, P- 5 MPa, Time - 4 h and D_2O-5 ml.

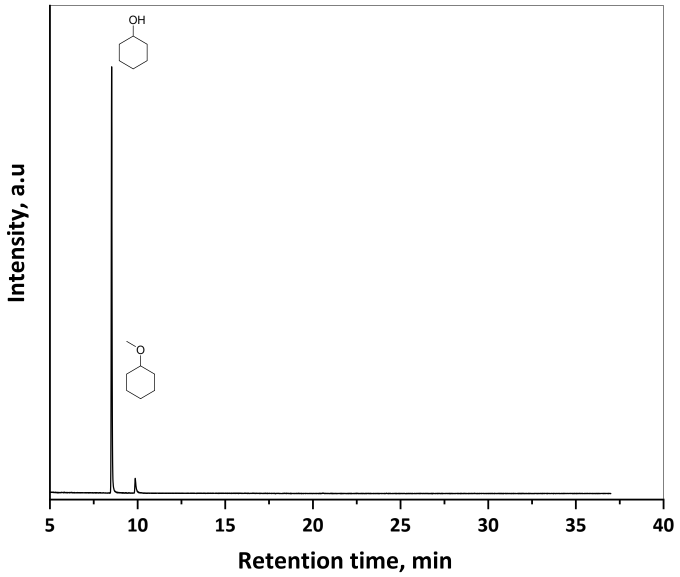

Figure S5: GC chromatogram of anisole hydrodeoxygenation over Ni_5Co_5-AC; Reaction condition: Catalyst- 100mg, T-180°C, P- 5 MPa, Time - 4 h and Water-5 ml.

Table S1: BET analysis of NiCo-AC done by N_2 adsorption-desorption isotherm.

Catalyst	S_{BET}, $m^2.g^{-1}$	V_p, $cm^3.g^{-1}$
AC	938	0.67
$Ni_{10}Co_0$-AC	791	0.15
Ni_8Co_2-AC	772	0.16
Ni_6Co_4-AC	797	0.18
Ni_5Co_5-AC	788	0.17
Ni_4Co_6-AC	784	0.14
Ni_2Co_8-AC	782	0.16
Ni_0Co_{10}-AC	791	0.20

Table S2: Effect of solvent on anisole hydrodeoxygenation.

Solvent	Conversion, %	Methoxy Cyclohexane, %	Cyclohexanol, %
Methanol	2	48	52
Ethanol	5	56	44
Iso-propanol	13	72	28
Iso-butanol	9	54	46
Water	100	5	95
Iso-propanol	100	88.2	11.8
**Reaction conditions:** Catalyst - Ni_5Co_5-AC, T- 180 °C, Anisole - 0.5 mmol, P - 5 MPa, Time - 4h			

Table S3: Reuse study of catalyst for anisole hydrodeoxygenation.

Cycle	Conversion, %	Methoxy Cyclohexane, %	Cyclohexanol, %
**1**	100	5	95
**2**	98.5	8	92.0
**3**	78.6	18.3	81.7
**4**	62	27.5	72.5
**Reaction conditions:** Catalyst - Ni_5Co_5-AC, T- 180 °C, Anisole - 0.5 mmol, P - 5 MPa, Time - 4h			

Time, min	Conversion, %	Methoxy Cyclohexane, %	Cyclohexanol, %
5	1.5	100	0
10	6.5	39	61
15	15	26	74
20	21	25	75
25	44	17	83
30	66	18	82
45	71	16	84
60	79	13	87
120	87	11	89
180	90	7.5	92.5
240	100	5	95
**Rxn conditions:** Catalyst - Ni_5Co_5-AC, T- 180 °C, Anisole - 0.5 mmol, P - 5 MPa			

Table S4: Impact of reaction time on anisole HDO and cyclohexanol selectivity.

According to the initial rate method, the rate of reaction can be given as

Rate = $\frac{-dc_{anisole}}{dt} = \frac{C_2 - C_1}{t_2 - t_1} = \frac{dc_{cychohexanol}}{dt}$ = K. $C_{cyclohexanol}^{n}$……………………(1)

Where K and n represent the rate constant and order of the reaction, respectively and n≠1.

$R_{0.5}$ and R_1 calculated by the initial rate equation.

$R_{0.5}$ =[$\frac{C_2 - C_1}{t_2 - t_1}$]$_{0.5\ mmol}$ = 0.294………………………(2)

R_1 =[$\frac{C_2 - C_1}{t_2 - t_1}$]$_{1\ mmol}$ = 0.662………………………(3)

Rate expression for 0.5 mmol

$R_{0.5}$= k. $C_{cyclohexanol}^{n}$……………………(4)

Rate expression for 1 mmol

R_1= k. $C_{cyclohexanol}^{n}$……………………….(5)

Dividing equation 5 by 4

$\frac{0.662}{0.294} = [\frac{1}{0.5}]^n$ ……………………….(6)

$2.25 = 2^n$

Taking log of equation 6

log 2.25 = n log 2

n = 1.125

Hence the order of the reaction is 1.125 and close to 1. The rate constant (K) was calculated by equation 5, and it was $1.1x10^{-2}.sec^{-1}$.
